# Non-gestational choriocarcinoma presenting as cervical lymphadenopathy in a male patient: a case report

**DOI:** 10.3389/fonc.2026.1874261

**Published:** 2026-06-29

**Authors:** Xiaoming Li, Daiying Gui, Chen Xie, Yunhong Xia

**Affiliations:** 1 Department of Oncology, The First Affiliated Hospital of Anhui Medical University, Hefei, Anhui, China; 2 Department of Oncology, Anhui Public Health Clinical Center, Hefei, Anhui, China

**Keywords:** case report, cervical lymphadenopathy, male, non-gestational choriocarcinoma, β-human chorionic gonadotropin

## Abstract

Non-gestational choriocarcinoma (NGCC) is a rare and highly aggressive malignancy in male patients and is frequently diagnosed at an advanced stage because of its atypical clinical manifestations. We report the case of a 38-year-old male patient who presented a unilateral cervical tumor without associated genitourinary symptoms. Histopathological examination combined with immunohistochemical analysis subsequently confirmed the diagnosis of NGCC. Radiologic assessment demonstrated extensive lymph node involvement without identification of a definite primary lesion. The patient initially showed a transient response following platinum-based chemotherapy, followed by disease progression during follow-up. This case highlights the importance of including NGCC in the differential diagnosis when male patients present with unexplained cervical lymphadenopathy, hemorrhagic and necrotic poorly differentiated tumors, and elevated β-human chorionic gonadotropin (β-hCG) levels. Early recognition together with comprehensive immunohistochemical evaluation and systemic imaging assessment is essential to reduce diagnostic delay.

## Introduction

Choriocarcinoma represents a highly aggressive form of trophoblastic tumor, characterized by rapid disease progression, early hematogenous spread, and markedly increased serum β-hCG levels (1). While this malignancy is most frequently associated with gestational events, NGCC in males is extremely rare and accounts for less than 0.1% of all testicular tumors (2).

In addition to the gonads, NGCC has been reported in several midline anatomical sites, including the mediastinum, retroperitoneum, and central nervous system, where it is generally classified as an extragonadal germ cell tumor (3–5). Due to its highly aggressive biological behavior and propensity for early systemic dissemination, the initial clinical presentation is frequently dominated by metastatic disease rather than symptoms originating from the primary lesion. Consequently, diagnosis is often delayed, particularly in patients lacking genitourinary manifestations.

Cervical lymphadenopathy as the initial manifestation of NGCC is particularly uncommon and can easily be misdiagnosed as malignant lymphoma or metastatic carcinoma of head and neck origin (6, 7). In this context, we report a rare case of male NGCC presenting primarily with cervical lymphadenopathy and discuss the diagnostic challenges and clinical implications.

## Case report

A 38-year-old man presented with a painless mass in the left cervical region that had persisted for more than one year. Physical examination revealed a firm, fixed mass in the left cervical region without overlying skin erythema or ulceration. The patient denied systemic symptoms such as fever, night sweats, or weight loss, and no genitourinary complaints. He had no relevant past medical history, family history of malignancy, or known genetic disorders. Initial evaluation was performed at an outside hospital. Serum β-hCG was markedly elevated at 4278 IU/L, whereas AFP was within the normal range. LDH was elevated during the disease course. PET-CT showed multiple enlarged lymph nodes with increased FDG uptake. Testicular ultrasonography revealed no definite testicular mass, although focal calcifications were noted in the left testis. Cervical ultrasonography demonstrated multiple enlarged lymph nodes in the left cervical region, with the largest measuring approximately 5.0 × 2.6 × 4.7 cm. Multiple enlarged retroperitoneal lymph nodes were observed, the largest measuring approximately 4.6 × 2.4 × 4.2 cm. To exclude hematologic malignancy, bone marrow aspiration and biopsy were performed, revealing hyperplastic bone marrow morphology with MF-0 reticulin staining and no evidence of myelofibrosis or primary hematologic malignancy. According to the external pathological report, the left cervical lymph node biopsy showed nests of highly atypical tumor cells with marked hemorrhage and extensive necrosis. Immunohistochemical analysis demonstrated positivity for pan-cytokeratin and β-hCG, with partial positivity for human placental lactogen (HPL), inhibin, SALL4, and GATA3, while ZBTB16, alpha-fetoprotein (AFP), and P63 were negative. The Ki-67 proliferation index was approximately 90%. These findings favored the diagnosis of non-gestational choriocarcinoma.

For further evaluation of disease extent, the patient was referred to our hospital. Repeat ultrasonography of the cervical and supraclavicular regions revealed multiple hypoechoic lymph nodes in the left cervical and supraclavicular areas with partially indistinct corticomedullary differentiation. Color Doppler flow imaging demonstrated punctate and linear vascular signals within the lymph nodes (**Figure 1**). Abdominal computed tomography (CT) showed enlarged retroperitoneal lymph nodes with a short-axis diameter of approximately 37 mm, raising suspicion for metastatic involvement in the context of the patient’s clinical history (**Figure 2**). Pelvic magnetic resonance imaging showed no obvious pelvic primary lesion (**Figure 3**). Scrotal magnetic resonance imaging demonstrated normal morphology of both testes without focal intratesticular mass or abnormal enhancement, and no definite primary testicular lesion was identified (**Figure 4**). No definite gonadal primary lesion was identified on the available imaging studies.

**Figure 1:**
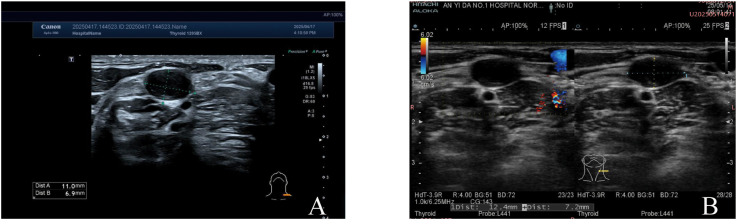
Ultrasonographic findings of cervical lymph nodes. **(A)** Gray-scale ultrasound showing an enlarged lymph node in the left cervical region. The measured lymph nodes in the representative images were approximately 11.0 × 6.9 mm and 12.4 × 7.2 mm. **(B)** Color Doppler ultrasound demonstrating internal vascular signals within the lymph node. Arrows indicate enlarged cervical lymph nodes and internal vascular signals.

**Figure 2:**
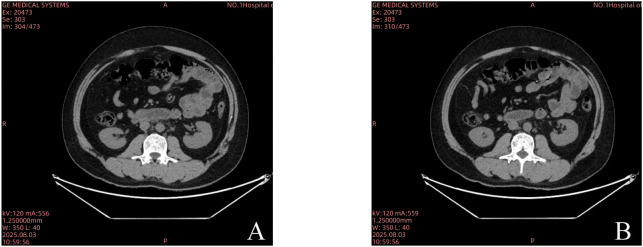
Abdominal CT showing retroperitoneal lymphadenopathy. **(A)** Non-contrast CT image showing multiple enlarged retroperitoneal lymph nodes. **(B)** Contrast-enhanced CT showing heterogeneous enhancement. The largest retroperitoneal lymph node measured approximately 4.6 × 2.4 × 4.2 cm. Arrows indicate enlarged retroperitoneal lymph nodes.

**Figure 3:**
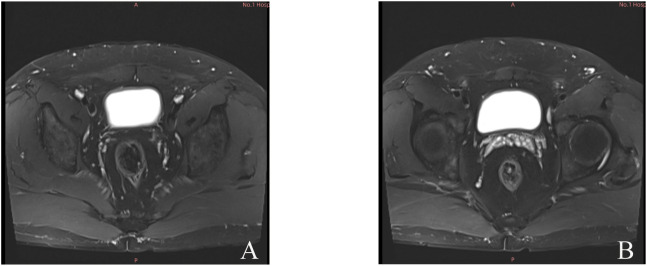
Pelvic magnetic resonance imaging.**(A)** Axial MRI of the pelvis. **(B)** Contrast-enhanced MRI showing no evident pelvic or genital primary tumor.

**Figure 4:**
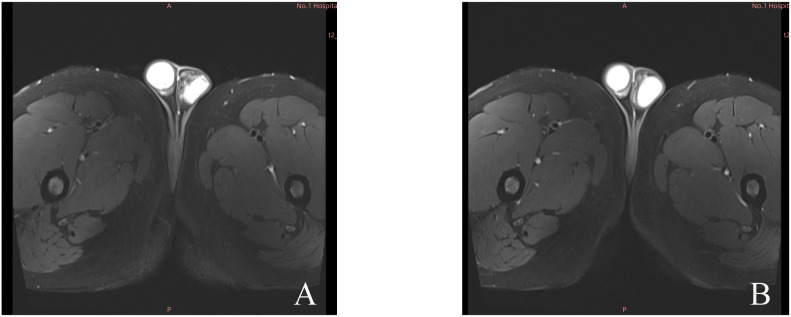
Scrotal magnetic resonance imaging. **(A, B)** Axial scrotal MRI showing normal morphology of both testes without a focal intratesticular mass or abnormal enhancement.

After non-gestational choriocarcinoma was suspected, the patient subsequently received platinum-based chemotherapy. In June 2024, he received the first cycle of EP chemotherapy consisting of etoposide 180 mg on days 1–3 and cisplatin 40 mg on days 1–3, after which the cervical mass markedly decreased in size. Four additional cycles of EP chemotherapy were administered from July to September 2024, consisting of etoposide 180 mg on days 1–3 and cisplatin 45 mg on days 1–3, and partial response was recorded in October 2024 based on serum β-hCG changes and follow-up imaging findings. Three further cycles of EP chemotherapy were administered from October to November 2024. The patient subsequently received multi-line chemotherapy because of disease progression and rising serum β-hCG levels. Follow-up CT in August 2025 showed retroperitoneal lymph node progression, and the regimen was modified in October 2025 because of disease progression and treatment-related adverse events. A concise clinical timeline of diagnosis, treatment, and follow-up is shown in **Figure 5**.

**Figure 5:**
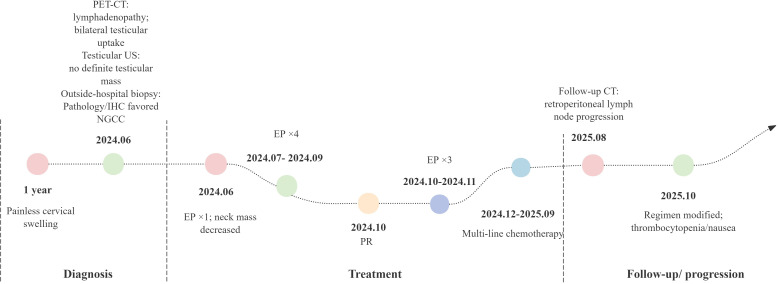
Clinical timeline of diagnosis, treatment, and follow-up. The timeline summarizes the key diagnostic work-up, treatment course, response assessment, and follow-up. EP, etoposide plus cisplatin; PR, partial response; NGCC, non-gestational choriocarcinoma; IHC, immunohistochemistry; US, ultrasonography.

## Discussion

NGCC in males is an exceedingly rare yet highly aggressive malignancy that is typically diagnosed at an advanced stage (8). Due to its nonspecific clinical manifestations and rapid metastatic progression, early recognition remains challenging. In the present case, the patient initially presented with isolated cervical lymphadenopathy in the absence of genitourinary symptoms, which significantly complicated the diagnostic process.

Previous studies consistently demonstrate that most male patients with NGCC present with metastatic disease at diagnosis, with approximately 70% exhibiting multi-organ dissemination, most commonly involving the lungs, brain, and lymph nodes (9). As summarized in **Table 1**, NGCC in males is characterized by markedly elevated serum β-hCG levels, early systemic dissemination, and a highly aggressive clinical course. Consistent with these findings, our patient had already developed extensive lymphatic metastases at presentation. However, this case is distinctive because the initial manifestation was painless cervical lymphadenopathy lasting for one year, without obvious genitourinary symptoms or a definite testicular mass on imaging. These features highlight the need to consider choriocarcinoma in male patients with persistent unexplained cervical lymphadenopathy, especially when serum β-hCG is elevated. The diagnostic challenge of NGCC largely stems from its nonspecific presentation and broad differential diagnosis. Clinically, it may mimic various malignancies, including lung cancer, lymphoma, and other metastatic tumors (23–26). In some cases, patients initially present with complications such as intracranial hemorrhage caused by brain metastases (27), while markedly elevated β-hCG levels may also lead to endocrine abnormalities, including gynecomastia and testicular atrophy (28). The absence of characteristic symptoms in our case further increased the risk of misdiagnosis. Therefore, NGCC should be considered in male patients presenting with unexplained cervical lymphadenopathy, particularly when accompanied by elevated serum β-hCG levels. The prolonged one-year interval before diagnosis may be related to the nonspecific initial presentation. Painless cervical lymphadenopathy is more commonly attributed to benign lymphadenopathy, lymphoma, or metastatic carcinoma from common primary sites. Because male NGCC is extremely rare and may occur without genitourinary symptoms, the diagnosis was not initially suspected.

**Table 1:** Clinical characteristics of previously reported cases of male non-gestational choriocarcinoma.

Case	Year	Age	Gender	β-hCG (IU/L)	Initial symptoms	Metastatic sites	Chemotherapy regimen	OS	Ref.
1	2021	32	male	200, 000	worsening fatigue, abdominal pain, non-bloody vomiting, and melena	Jejunal, Lung	Cisplatin, ifosfamide, etoposide	36 days	(10)
2	2022	24	male	104, 712	pleuritic chest pain and back pain	Lung, abdomen, pelvis	VIP (V=etoposide, I=ifosfamide, P=cisplatin)	live	(11)
3	2023	40	male	–	fever, cough, dizziness, melena, and severe anemia	Lung, small bowel,	BEP (B=bleomycin, E=etoposide, P=cisplatin)	4 months	(12)
4	2019	30	male	40, 453	dyspnea, right-sided chest pain	Lung, gastric	BEP (B=bleomycin, E=etoposide, P=cisplatin)	–	(13)
5	2018	37	male	278, 800	aching groin pain	Dermatological, brain, gastrointestinal	BEP (B=bleomycin, E=etoposide, P=cisplatin)	–	(14)
6	2019	26	male	–	back pain, dyspnea, high fever	Lung	BEP (B=bleomycin, E=etoposide, P=cisplatin)	6 months	(15)
7	2022	40	male	–	left ptosis with a lateral, downward deviation of the left eye	Brain	BEP (B=bleomycin, E=etoposide, P=cisplatin)	6 months	(16)
8	2015	18	male	355, 000	headache, drowsiness, vomiting, confusion	brain	BEP (B=bleomycin, E=etoposide, P=cisplatin)	–	(17)
9	2025	67	male	5, 514	cough, expectoration, blood in the sputum	Lung	cisplatin, etoposide, paclitaxel, and sintilimab	CR at 12months	(18)
10	2016	67	male	–	left pulmonary mass	Lung	BEP (B=bleomycin, E=etoposide, P=cisplatin), vincristine, elemene	CR at 13months	(19)
11	2013	68	male		melena	sigmoid colon	mFOLFOX6, bevacizumab	9 months	(20)
12	2015	20	male	163, 000	increasing right testicular swelling	Lung	BEP (B=bleomycin, E=etoposide, P=cisplatin)	live	(21)
13	2018	18	male	10, 000	cutaneous mass at the left back	cutaneous	BEP (B=bleomycin, E=etoposide, P=cisplatin)	3 months	(22)

OS refers to the interval from diagnosis to death or last follow-up.

Cervical lymph node involvement as the initial manifestation of NGCC is extremely rare. Only sporadic cases have been reported, including presentations as cervical masses or head and neck lesions. Ahmadi et al. reported a neck mass as the first presentation of testicular choriocarcinoma (6), Payne et al. described an extragonadal case with tonsillar presentation (7), and Senkal et al. reported metastatic choriocarcinoma presenting as a neck mass (29). Compared with these cases, the present patient had a prolonged one-year history of painless cervical lymphadenopathy and no definite testicular mass on available imaging. One possible mechanism is that tumor cells disseminate via the thoracic duct to the supraclavicular and cervical lymphatic chains. Consistent with this hypothesis, the present case exhibited extensive lymphatic metastases at diagnosis, suggesting that systemic spread had already occurred prior to the onset of overt clinical symptoms.

Histopathological evaluation combined with immunohistochemical analysis remains essential for establishing a definitive diagnosis. However, in small biopsy specimens, extensive hemorrhage and necrosis may obscure trophoblastic differentiation, increasing the likelihood of misdiagnosis (7). In this case, the tumor exhibited marked cytological atypia with extensive hemorrhage and necrosis. Immunohistochemistry demonstrated positivity for β-hCG and human placental lactogen, with partial expression of SALL4, supporting trophoblastic differentiation. Negative staining for AFP and LCA excluded yolk sac tumor and hematolymphoid malignancies, respectively. The high Ki-67 proliferation index further indicated an aggressive biological phenotype. Another limitation is that the cervical lymph node biopsy was performed at an outside hospital, and the original pathological slides, paraffin blocks, and higher-magnification photomicrographs were unavailable for further review. Therefore, the pathological interpretation was based on the available external pathological report.

Radiological evaluation plays a critical role in disease staging and identifying the potential primary tumor. However, imaging findings in germ cell tumors are often nonspecific and may mimic lymphoma or metastatic carcinoma (30). In this case, no definite testicular lesion was identified despite comprehensive imaging. This raises the possibility of a “burned-out” testicular tumor or an extragonadal origin. Previous studies have suggested that the primary tumor may undergo spontaneous regression, leaving minimal or undetectable residual change (31, 32). However, an occult or burned-out testicular tumor could not be completely excluded by imaging alone and was therefore acknowledged as an important diagnostic limitation. Therefore, even in the absence of a detectable primary lesion, the diagnosis should rely on integrated clinical, radiological, and pathological evaluation.

From a therapeutic perspective, NGCC is generally sensitive to platinum-based chemotherapy regimens such as EP or BEP in the initial phase (33, 34). However, durable responses remain limited. In the present case, although the patient initially responded to EP chemotherapy, rapid disease progression subsequently occurred, reflecting early chemoresistance. This clinical course is consistent with the aggressive nature of NGCC. While salvage regimens and high-dose chemotherapy may provide benefit in selected patients (35–38), their overall efficacy remains limited. These findings underscore the need for improved therapeutic strategies.

In summary, this case highlights several clinically important features of NGCC, including an extremely rare initial presentation with isolated cervical lymphadenopathy, absence of an identifiable primary lesion, and rapid disease progression despite an initial chemotherapy response. These findings emphasize that NGCC should be considered in the differential diagnosis of unexplained cervical lymphadenopathy in male patients. Early recognition and prompt pathological confirmation are critical for improving clinical outcomes.

## Conclusion

NGCC is a rare but highly aggressive malignancy that may initially present as unexplained cervical lymphadenopathy in male patients. When biopsy reveals a hemorrhagic and necrotic poorly differentiated tumor, especially in the presence of elevated serum β-hCG, NGCC should be included in the differential diagnosis. Early pathological confirmation, comprehensive immunohistochemical assessment, and systemic imaging evaluation are essential to reduce diagnostic delay and guide timely treatment.

## Data Availability

The original contributions presented in the study are included in the article/supplementary material. Further inquiries can be directed to the corresponding author.
